# Effect of Warfarin on Lifespan and Oxidative Stress Tolerance of *Drosophila melanogaster*

**DOI:** 10.3390/ijms26104808

**Published:** 2025-05-17

**Authors:** Anna Lavrenova, Oleg Klychnikov, Vitaliy Ioutsi, Igor Rodin, Oksana Luneva, Lidia Nefedova

**Affiliations:** 1 Department of Genetics, Faculty of Biology, M. V. Lomonosov Moscow State University, 119234 Moscow, Russia; anigd_1999@mail.ru; 2 Department of Biochemistry, Faculty of Biology, M. V. Lomonosov Moscow State University, 119234 Moscow, Russia; oklych@yahoo.co.uk; 3 The National Medical Research Center for Endocrinology, 117292 Moscow, Russia; outsi.vitalij@endocrincentr.ru; 4 Department of Analytical Chemistry, Faculty of Chemistry, M. V. Lomonosov Moscow State University, 119991 Moscow, Russia; igorrodin@yandex.ru; 5 Lomonosov Institute of Fine Chemical Technologies, Russian Technological University, 119571 Moscow, Russia; 6 Department of Biophysics, Faculty of Biology, M. V. Lomonosov Moscow State University, 119234 Moscow, Russia; oluneva@yandex.ru

**Keywords:** drosophila, vitamin K, warfarin

## Abstract

In vertebrates, vitamin K is a cofactor for the gamma-glutamyl carboxylase (GGCX) involved in the carboxylation of glutamic acid residues. During the vitamin K cycle, vitamin K is oxidised by GGCX, and then reduced by vitamin K epoxide reductase (VKOR), which is inhibited by the synthetic coumarin warfarin. GGCX and VKOR are present in *Drosophila melanogaster*, but the existence of a vitamin K cycle remains unproven. Semi-lethal concentrations (LC_50_) of K_3_, menadione sodium bisulfite (MSB), and warfarin to neutralise the negative effect of MSB were selected for the Drosophila cultivation medium. LC-MS analysis was used for vitamin K measurement in flies’ extracts. The EPR method and RT-PCR were used for ROS level measurement and gene transcription assessment, respectively. The LC_50_ of MSB in the medium resulted in a more than 20-fold increase in endogenous K_2_ in flies, demonstrating the mechanism of K_3_-to-K_2_ conversion. Administration of 1 mM warfarin in the medium with MSB completely neutralised its negative effect on viability. Developed flies had decreased K_2_ level, confirming the existence of a vitamin K cycle, and both reduced ROS level and *hsp22* gene transcription. The biochemical pathways affected by elevated K_2_ concentrations involves both elements of the vitamin K cycle and the adaptive mitochondrial antioxidant system.

## 1. Introduction

Vitamin K is one of the most important compounds for most living organisms [1]. In plants and bacteria, vitamin K acts as an electron carrier in the electron transport chain [2,3]. In humans and other vertebrates, vitamin K is a cofactor for the enzyme gamma-glutamyl carboxylase (GGCX), which is involved in the carboxylation of protein domains containing glutamic acid residue (Glu). The enzymatic reaction of glutamate γ-carboxylation forms γ-carboxyglutamate (Gla), capable of binding calcium with high affinity. The Gla-rich domains were initially identified in prothrombin and some other proteins of the blood coagulation system [4]. However, in recent years, Gla proteins with different functions and different cellular and tissue localizations have been identified. Currently, 17 vitamin K-dependent Gla proteins have been described that play key roles in regulating physiological processes in humans [5,6]. Seven of them perform their functions in the liver: these are the coagulation factors II, VII, IX, and X and the anticoagulant proteins C, S, and Z. Six others are involved in various physiological and pathological processes in extrahepatic tissues: OS, MGP, Gas6, GRP, periostin, and periostin-like protein. The functions of the remaining four proteins—proline-rich Gla 1, proline-rich Gla 2, transmembrane Gla 3, and transmembrane Gla 4—are poorly characterised. A common feature of all these proteins is the presence of Gla domains [7].

During the carboxylation reaction of Gla proteins, vitamin K is oxidised by GGCX to epoxide, and then reduced to quinone and hydroquinone by another enzyme—vitamin K epoxide reductase (VKOR). These sequential reactions of oxidation and reduction in vitamin K are called the vitamin K cycle [8].

Blocking the vitamin K cycle leads to impaired blood clotting and, as a result, death [9]. In mammals, plant-originating and synthetic coumarins, such as warfarin, act as anticoagulants causing the accumulation of non-carboxylated Gla proteins in the coagulation system [10]. Like all coumarins, warfarin acts as an indirect vitamin K cycle blocker, i.e., it does not bind to GGCX and does not affect the gamma-carboxylation reaction, but inhibits a reduction in vitamin K epoxide via VKOR. This is a cause of haemorrhage and death in vertebrates [9]. The mechanism of inhibition has been hypothesised, but the process has not been fully elucidated. Some studies [11] show that warfarin acts on VKOR by a competitive inhibition mechanism, while other data [12] show that warfarin inhibits VKOR by a non-competitive inhibition mechanism. One of the most recently published studies [9] showed that warfarin-binding sites could be located both inside and outside the vitamin K binding site, which may explain the difference in the mechanism of inhibition.

There are only two non-polar forms of vitamin K in nature, K_1_ and K_2_; the other forms of vitamin K, K_3_–K_7_, are synthetic analogues of the natural vitamin. In medical practice, the water-soluble salt of vitamin K_3_—menadione sodium bisulfite (hereinafter MSB)—is most commonly used to improve blood coagulation by increasing the synthesis of coagulation factors II, VII, IX, and X; it is also used in the treatment of vitamin K deficiency [13].

In addition to its important role in blood coagulation, MSB has long been used in various studies to induce oxidative stress [14,15,16,17]. It has been shown that MSB can act as an inducer of oxidative stress, as it can stimulate the production of reactive oxygen species (ROS) and leads to apoptosis [18,19]. The ability of vitamin K to generate ROS is used in medicine to treat tumours [20].

At the same time, vitamin K is a powerful antioxidant. Vitamin K not only increases the number of surviving cells during oxidative stress, but also reduces the amount of ROS in cells [21]. It was shown that the antioxidant activity of vitamin K is much greater than that of alpha-tocopherol and ubiquinone [22]. Moreover, vitamin K_2_ can be used to synthesise ATP instead of ubiquinone during electron transfer, which helps maintain mitochondrial function [23].

Given its antioxidant properties, vitamin K has recently been used not only as a drug that promotes blood clotting, but also in the treatment of neurodegenerative diseases such as Alzheimer’s and Parkinson’s. In the case of Alzheimer’s, vitamin K appears to act as an anti-inflammatory factor, having an antioxidant effect [24]; in the case of Parkinson’s disease, it takes on the role of an additional electron carrier, thereby helping to reduce ATP deficiency in patients [25].

The enzymes involved in the vitamin K cycle (GGCX and VKOR) are found primarily in vertebrates and also in arthropods. The genes for the enzymes GGCX and VKOR are present in *Drosophila melanogaster*, but there is no evidence for a vitamin K cycle [7,26,27,28]. It has been shown that *D. melanogaster* GGCX is capable of effectively performing gamma-carboxylation of prothrombin and human coagulation factor IX [26], indicating that substrate recognition appears to be highly conserved between humans and Drosophila.

Interestingly, knocking out the *GGCX* gene, *GC*, in *D. melanogaster* does not affect the viability of the flies [26]. This makes fruit flies a convenient model for detailed studies of vitamin K metabolism compared to mammals. The demonstration of the presence of a vitamin K cycle in *D. melanogaster* will allow the use of this classic genetic model to study the molecular genetic mechanisms of action of drugs that affect the vitamin K cycle in vertebrates. Given the fact that *D. melanogaster* has active GGCX, but no classical Gla proteins have been described, the search for potential targets of gamma-carboxylation will expand our understanding of the functional capabilities and mechanisms of GGCX evolution in animals.

In the present research, we have shown for the first time that warfarin does not have a negative effect on the viability of *D. melanogaster*, and that warfarin neutralises the lethal effect of high doses of vitamin K by reducing the level of oxidative stress. Taken together, these data suggest that a vitamin K cycle does exist in Drosophila and that this animal model is suitable for a detailed in vivo study of the mechanisms involved in the functioning of the vitamin K cycle.

## 2. Results

### 2.1. Concentration Effect of MSB and Warfarin on Survival of D. melanogaster

Previously, a manuscript by Jordan et al. [16] described a dependence of the lifespan of *D. melanogaster* imago on the MSB in the nutrient medium: higher concentrations of MSB (greater than 3 mM) caused a significant shortening of the lifespan of adult flies, while low doses slightly increased the average life expectancy. In the present work, we tested MSB’s effect on egg-to-imago development and the role of the vitamin K cycle blocker warfarin in this process.

In our experiments, we have confirmed that the minimum lethal concentration of MSB in a medium for Drosophila is 10 mM. All larvae that developed on this medium died and did not develop into imago. Interestingly, at high doses of MSB (10 mM), some of the larvae showed food avoidance behaviour—crawling out of the agar on the walls of test tubes. We suppose that these larvae were most likely dying from premature exit from the nutrient medium rather than from the toxic effect of MSB. To test this, we transferred these larvae to a medium without MSB, where they continued to develop normally by burrowing into the nutrient agar. We associate this behaviour with the negative chemotaxis of the larvae towards high doses of MSB.

**Figure 1:**
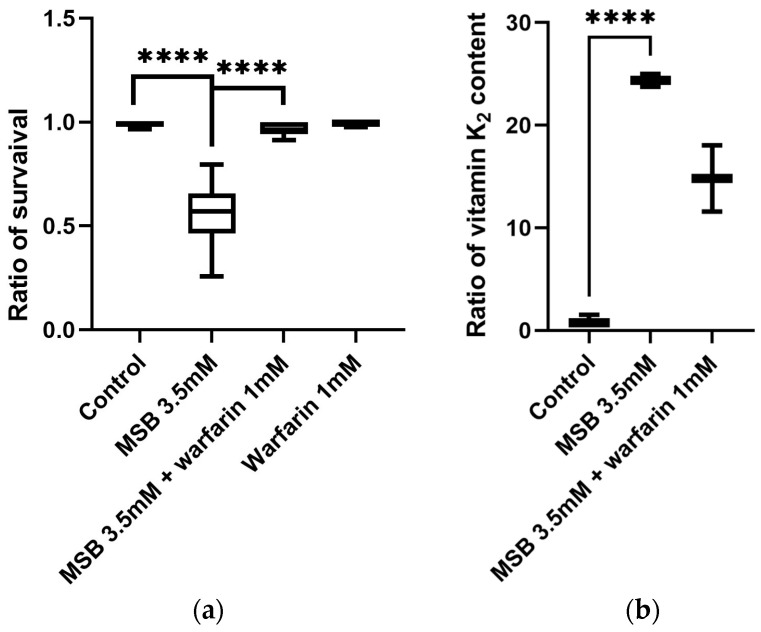
Effect of externally applied MSB and warfarin on the larval survival rate and vitamin K_2_ content in adult *D. melanogaster*. (**a**) Relative number of fruit flies developed on standard medium (control) and media supplemented with MSB, or MSB plus warfarin, or warfarin, normalised to the total number of pupae; (**b**) relative amount of vitamin K_2_ in adult *D. melanogaster* grown on standard medium and media supplemented with MSB or MSB plus warfarin, determined by LS-MS. Vitamin K levels are normalised to the control. Box plot constructed using median values with minimum and maximum vitamin K_2_ values. Statistical analysis of data using paired two-tailed Student’s *t*-test (**** *p* < 0.0001).

We also determined the half-lethal concentration of MSB at which half of the larvae would survive and develop into adults—3.5 mM (Figure 1a). We tested the effect of warfarin on fly viability in the range from 0.1 to 10 mM (Supplementary Table S1). The concentration of warfarin we used was equivalent to lethal doses for mammals. Surprisingly, warfarin in this range had no apparent effect on larval behaviour and viability.

We also tested the effect of warfarin against a lethal dose of vitamin K. When warfarin was added to the medium containing 10 mM of MSB, it was able to neutralise the effect of MSB at the minimum concentration of 1 mM (Supplementary Table S1). Therefore, this concentration of warfarin was used in all subsequent experiments. Not surprisingly, warfarin at a concentration of 1 mM in the medium completely neutralised the effect of MSB at a half-lethal concentration of 3.5 mM (Figure 1a). Based on these results, we used MSB and warfarin at these concentrations (3.5 mM and 1 mM, respectively) in further experiments.

### 2.2. Vitamin K Content in D. melanogaster

Having demonstrated the effect of MSB and warfarin on *D. melanogaster* viability, we decided to determine vitamin K level in flies to answer the following questions: is MSB converted to vitamin K_2_ and can the vitamin K cycle be blocked in the similar way as in mammals?

As no one had previously reported on the vitamin K content of insects, we needed to develop a protocol for the extraction and LC-MS measurement of vitamin K. We started by using control flies to determine whether vitamin K is normally present in Drosophila and how their vitamin K levels relate to vitamin K levels in mammals. The first step in developing a quantitative protocol was to select an organic solvent that would effectively extract our target analyte from Drosophila. Several individual solvents were tested for their efficiency in extracting vitamin K: methanol, methyl tert-butyl ether (MTBE), ethyl acetate, and a hexane–isopropanol mixture (1:1 by volume). We also optimised the conditions for chromatography–mass spectrometry analysis of vitamin K using multiple reaction monitoring (MRM). Our experiments showed that in *D. melanogaster* the most effective extractants were methanol and MTBE (Supplementary Table S2).

The reproducibility of the developed protocol was also tested. For this purpose, we performed the extraction protocol on three independent samples. Completeness was tested by performing the methanol extraction protocol first, followed by MTBE extraction. The first extraction gave a vitamin K_2_ level of 0.139 ± 0.003 ng/g of flies (w/fw). The use of an additional extraction protocol yielded an additional 0.0119 ± 0.0006 ng/g (w/fw) of vitamin K_2_, corresponding to 8.6% extra. Since MTBE is more toxic to personnel and the environment than methanol, we decided to continue the experimental procedure with methanol with an increased number of vitamin K extraction steps (seven times, 1 mL of methanol each time). Based on this protocol, we determined the vitamin K_2_ content in control flies developing on the standard medium—0.26 ± 0.07 ng/g (w/fw) of flies.

To study the effects of MSB and warfarin on vitamin K levels in Drosophila, flies were reared from the egg to the pupal stage on media containing 3.5 mM MSB and 3.5 mM MSB plus 1 mM of warfarin (Figure 1b). After pupal emergence, flies were collected and vitamin K was extracted and quantified according to the LC-MS protocol. Analysis of MRM transitions showed that, in adult Drosophila developing on 3.5 mM of MSB, the level of vitamin K_2_ increased by more than 20 times compared to the control, reaching 6.34 ± 0.17 ng/g (w/fw). When warfarin and MSB were applied together, the K_2_ level decreased by a factor of 2 compared to the sample without an inhibitor—3.85 ± 0.85 ng/g (w/fw).

### 2.3. ROS Determination in Adult D. melanogaster Treated with High Doses of MSB

Since we observed a change in Drosophila survival at high MSB concentrations, we decided to verify if this effect was associated with a change in ROS levels. The electron paramagnetic resonance (EPR) technique is a conventional method for measuring the production of short-lived inorganic radicals in cell, which is often described as the «gold standard» for the detection and characterisation of radicals in biological systems [29]. In our experiments, non-specific spin probes based on cyclic hydroxylamine were used to reveal tentative changes in the overall oxidative state of drosophila cells. To assess the oxidative status in the samples, we used 1-hydroxy-4-isobutyramido-2,2,6,6-tetramethylpiperidinium chloride (TMT-H), which belongs to the group of cyclic hydroxylamines and has a low ability to penetrate the lipid bilayer [30]. Cyclic hydroxylamines can be oxidised by various ROS, transition metal ions, and some haem-containing enzymes. In all cases, the reaction product is always the same—the nitroxyl radical, which gives a characteristic EPR signal [30,31]. In the case of cyclic hydroxylamines, the EPR spectrum does not allow the nature of the ROS source to be determined, but the overall oxidising capacity in a sample can be inferred from the intensity of the EPR spectrum. For this, the intensity value of the central component of the TMT-H EPR spectrum in arbitrary units was used to assess the oxidation state of the samples. This parameter reflects the number of paramagnetic centres formed during the interaction of TMT-H with ROS.

**Figure 2:**
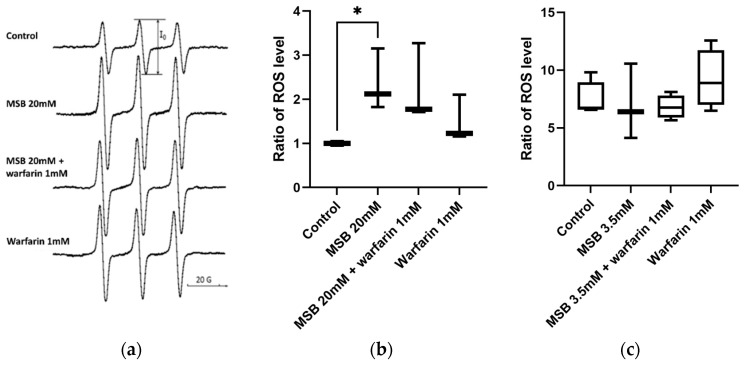
Determination of ROS levels in homogenates of adult *D. melanogaster* treated with MSB, MSB plus warfarin, or warfarin, by EPR technique. (**a**) Representative EPR spectra of TMT-H of control flies and flies treated with MSB and warfarin; (**b**) acute stress conditions (adult flies, 24 h of treatment in a medium: 20 mM MSB, 20 mM MSB plus 1 mM warfarin, or 1 mM warfarin). Relative values of ROS are based on the average intensity of the central component (I_0_) of the EPR spectrum of TMT-H. Statistical analysis of data using paired two-tailed Student’s *t*-test (* *p* < 0.05); (**c**) chronic stress conditions (flies developing from egg to pupal stage on media: 3.5 mM MSB, 3.5 mM MSB plus 1 mM warfarin, or 1 mM warfarin). Quantitative analysis of relative values of ROS based on the average intensity of the central component (I_0_) of the EPR spectrum of TMT-H.

Therefore, in this experiment, we decided to use 2-day-old adult flies, which were grown on standard medium and then placed on standard medium with the tested substances for 24 h as an acute stressor: (1) no substances—control, (2) 20 mM MSB, (3) 20 mM MSB and 1 mM warfarin, and (4) 1 mM warfarin. Analysis of the intensity of the central component (I_0_) of the TMT-H EPR spectrum (Figure 2a) showed that extracts from flies treated with MSB showed at least a 2-fold increase in oxidative status in homogenates compared to the control, and that the application of warfarin tended to decrease this level (Figure 2b). We also measured ROS levels in flies developing from the egg to the pupal stage on media containing MSB and warfarin, but found no statistically significant differences in ROS levels (Figure 2c).

### 2.4. Gamma-Glutamyl Carboxylase Gene GC Expression in Adult D. melanogaster in Response to MSB and Warfarin

**Figure 3:**
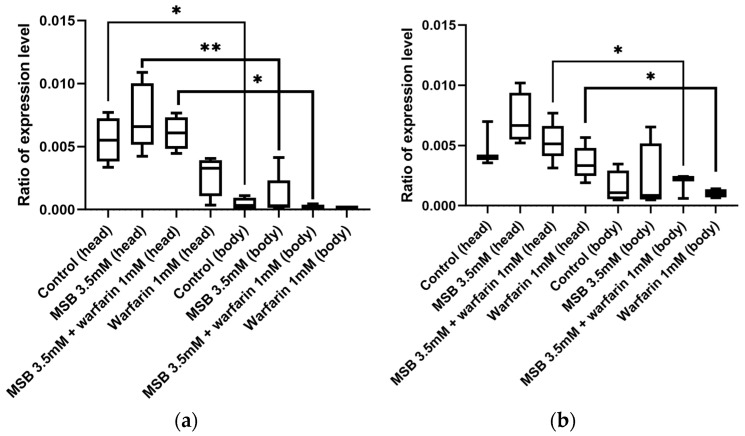
The transcription level of the *GC* gene in the heads and bodies of *D. melanogaster* imago developed on media with MSB and warfarin (* *p* < 0.05, ** *p* < 0.01, Mann–Whitney U test): (**a**) females; (**b**) males.

As the gamma-glutamyl carboxylase (GGCX) is a key enzyme in the vitamin K cycle, we decided to investigate if the level of its mRNA will correlate with the amount of externally applied MSB. According to the analysis of the *D. melanogaster* transcriptome in the FlyBase database (https://flybase.org/reports/FBgn0035245#expression, accessed on 23 January 2024), the *GC* gene is mainly expressed in the central nervous system, with expression levels in the brain two orders of magnitude higher than in other tissues. Based on this, we analysed expression in the heads and bodies of adult flies of both sexes separately. We observed an increased level of *GC* gene expression in the heads of flies, but low *GC* expression was observed in the bodies (Figure 3), which is consistent with the FlyBase data. *GC* expression levels do not change significantly when MSB or warfarin are added to the diet. Thus, *GC* transcription is not feedback-dependent on vitamin K levels.

### 2.5. Expression Level of the Oxidative Stress Marker Gene hsp22 in D. melanogaster in Response to MSB and Warfarin

In the present study, elevated levels of ROS were detected in *D. melanogaster* by EPR under acute stress. We decided to test a hypothesis of whether a genetic marker of mitochondrial stress, heat shock protein 22 (Hsp22), would also be affected. Hsp22 is a molecular chaperone that functions in the mitochondrial matrix and is activated by oxidative stress. Increased expression of *hsp22* has been shown to be positively correlated with increased adult lifespan and stress resistance [32].

**Figure 4:**
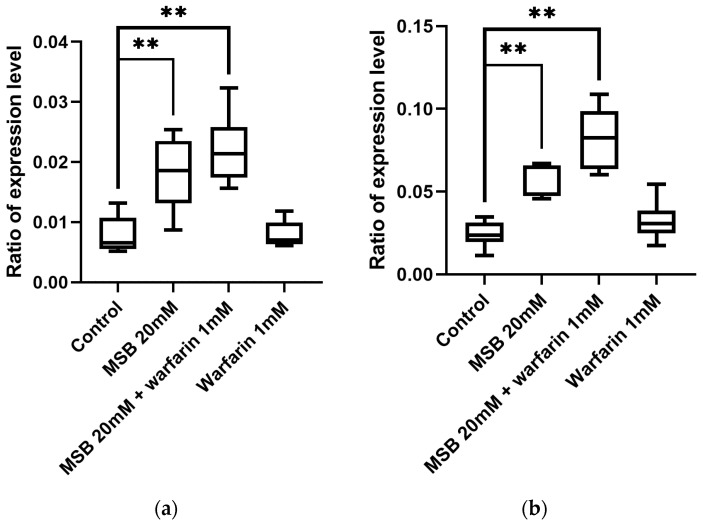
Level of *hsp22* gene expression in *D. melanogaster* adult flies after 24 h of incubation on a medium with MSB and/or warfarin (** *p* < 0.01, Mann–Whitney test): (**a**) females; (**b**) males.

Quantitative measurements of the level of *hsp22* transcription by qPCR (Figure 4) demonstrated that it increased in both males and females that were treated with high doses of MSB and MSB in combination with warfarin. This finding is consistent with the data of ROS level increase obtained by the EPR method (Figure 2b).

Then, we studied the effect of warfarin and MSB on flies developed on a medium with different concentrations of MSB and warfarin (chronic stress conditions). Since in these conditions we found no statistically significant differences in ROS levels by EPR, we looked at the levels of gene expression in heads and bodies separately for males and females.

**Figure 5:**
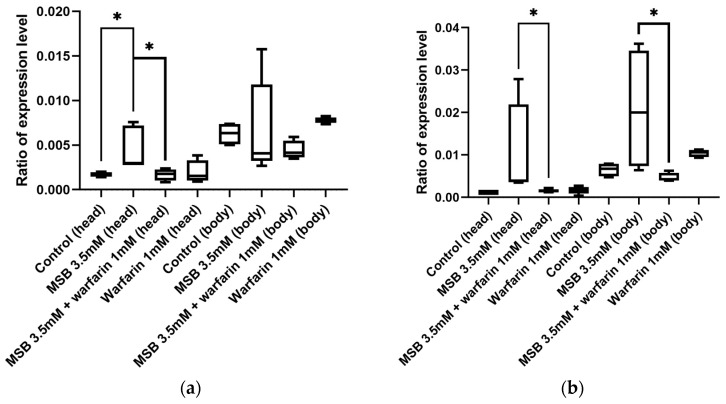
The level of expression of *hsp22* in the heads and bodies of *D. melanogaster* adults on media with MSB and warfarin (* *p* < 0.05, Mann–Whitney test): (**a**) females; (**b**) males.

As shown in Figure 5, the expression level of *hsp22* in the heads of flies grown on a medium with a concentration of MSB of 3.5 mM was significantly increased. However, when the medium contained MSB and warfarin, the expression level of the *hsp22* gene decreased to a value similar to the control. In the body, a decrease in *hsp22* expression was only seen in males that were grown on warfarin along with MSB. It is noteworthy that, compared with high doses of MSB (20 mM) used in acute stress, the effect of 3.5 mM MSB on *hsp22* transcription is neutralised by warfarin. However, changes in *hsp22* transcription levels are not as significant (evidenced by *p*-values) as in acute stress. This can explain the EPR results under chronic stress conditions.

## 3. Discussion

In this work, we confirmed that concentrations of MSB (vitamin K_3_) above 3 mM in a nutrient medium not only affect the lifespans of adult Drosophila, as previously shown [16], but also significantly reduce larval survival. To investigate whether this effect involves the conversion of K_3_ to K_2_, we developed a quantitative protocol for the extraction of vitamin K_2_ from adult Drosophila and its detection by LC-MS (MRM) analysis. Based on this protocol, we determined for the first time the concentration of vitamin K_2_ in adult Drosophila flies: 0.26 ± 0.07 ng/g (w/fw). We also estimated the average concentration of vitamin K_2_ in the haemolymph of the flies, taking into account the following considerations: one gram of Drosophila contains 1000–1200 flies, and the sex ratio was approximately 1:1. From the literature, the average haemolymph volume of a fly was estimated to be 56 ± 8 nL for females and 24 ± 2 nL for males [33]. This gives an average of 40 nL, and therefore an average vitamin K_2_ content in the haemolymph of *D. melanogaster* of 6 ng/mL. This level of vitamin K_2_ in Drosophila haemolymph is comparable to that in mammalian blood plasma [34] and may indicate either a convergent or parallel evolution of the biochemical composition of these biological fluids in insects and vertebrates.

We also show that in flies with an external supply of vitamin K_3_ (MSB) in the nutrient medium, the level of endogenous vitamin K_2_ increases more than 20-fold (6.34 ± 0.17 ng/g w/fw). This shows that the biochemical mechanism of K_3_ to K_2_ conversion, including the prenyltransferase enzyme described previously for rats [35], can take place in insects. In a manuscript by Hirota et al. [35], the authors used deuterated forms of vitamin K in an APCI-LC-MS/MS experiment. By direct measurements of the products, they proposed a detailed mechanism of K_1_-to-K_2_ conversion. This study demonstrates that K_1_ is converted to K_3_ after the cleavage of the phytil chain in the enterocytes of the intestine. Then, K_3_ as a transport form can be delivered via the blood and lymphatic systems to a target tissue, in which it should be reduced to hydroquinone converted to K_2_ by prenyltransferase (UBIA1_DROME). The localisation of this enzyme according to the Uniprot database (https://www.uniprot.org/uniprotkb/Q9V3R8/entry, accessed on 12 March 2024) is detected in mitochondria, EPR, and the Golgi apparatus. The details of the mechanism of K_3_-to-K_2_ conversion in Drosophila need to be further investigated.

Since the conversion of K_3_ to K_2_ by the UbiA prenyltransferase-containing domain takes place in the mitochondria [36], and vitamin K can be used as an alternative carrier in the electron transport chain due to its quinone nature, we tested the hypothesis that the mitochondria can be a target organelle.

The levels of ROS measured by EPR show that the high levels of vitamin K may indeed increase ROS production, but the effect of warfarin on ROS levels showed a trend towards a decrease, which needs to be further investigated. We achieved the greatest effect with acute stress, but not with chronic stress. We hypothesised that because MSB leads to larval mortality, flies that hatched may have already adapted to MSB at the larval stage, so their ROS levels were not elevated or elevated at an undetectable level.

We also tested a marker of oxidative stress, *hsp22*. We observed a statistically significant increase in the expression level of this gene in imagoes reared on different concentrations of MSB. When flies were given warfarin in addition to 3.5 mM MSB during larval development, a decrease in the expression level of *hsp22* was observed. This effect was more pronounced in the head, which correlates with high-level ROS production in the brain [37]. As for ROS production, *hsp22* transcription depends on MSB dose: a higher dose has a greater effect on both ROS production and oxidative stress gene transcription.

It should be noted that, using EPR, we discovered a reliable change in oxidative state, but to identify particular ROS and locations of the reactions in cells, we are planning to perform further experiments. In particular, we are going to use specific spin traps for superoxide anion and hydroxyl radicals (DEPMPO and DMPO) separately in heads and bodies.

We also tested the expression level of the key component of the vitamin K cycle—GGCX—which uses vitamin K as a cofactor in the gamma-carboxylation of glutamic acid. In addition to vertebrates, GGCX has also been found in the marine snail *Conus* [28]. There, Gla protein is present in snail venom peptides. Biochemical experiments have shown that the post-translational conversion of glutamate to Gla in Conus peptides has the same general requirements as γ-carboxylation in mammals [38]. Nevertheless, the overall topology of the Conus, Drosophila, and human GGCX enzymes is similar. This high degree of similarity is consistent with the hypothesis of a common evolutionary origin of these enzymes [7]. Previously, a GGCX activity homologous to human GGCX was found in Drosophila [27] and biochemically investigated. A vitamin K-dependent activity was found. In [26], it was shown that the Drosophila enzyme recognises propeptides of human prothrombin and factor IX and that substrate recognition appears to be highly conserved. However, inactivation of the GGCX gene in Drosophila has no visible effect on growth and fertility under laboratory conditions. The GGCX gene in Drosophila is transcribed at very low levels, making it difficult to study its expression, and is not induced in response to stress, according to the FlyBase database. According to our data, the level of this transcription was not affected by either the increased levels of vitamin K or the use of warfarin in any combination. This may be due to the fact that the high concentration of vitamin K_2_ is localised in a specific compartment and may not be toxic to the whole cell, but to a specific organelle.

The application of warfarin together with high concentrations of vitamin K_3_ neutralised its toxic effects. Quantitative determination of vitamin K_2_ levels in fly homogenate showed an approximately 2-fold decrease in vitamin levels in the presence of 1 mM warfarin. Such a decrease in the quinone form of vitamin K_2_ may indicate the accumulation of the oxidised epoxy form, as well as a decrease in the reduced K_2_ consumed in metabolism. These results provide indirect evidence for the existence of a vitamin K cycle in *D. melanogaster* similar to that previously described in mammals [8]. Surprisingly, even that high dose of warfarin did not reduce the vitamin K_2_ level to the control.

In mammals, warfarin is used to block VKOR, preventing the reduction of vitamin K from its epoxy form and resulting in downstream inhibition of blood coagulation mechanism [9]. In higher concentrations, warfarin is used for pest control, and the LC_50_ for mice, rats, cats, and dogs is 1.5–6 mg/kg (https://www.cdc.gov/niosh/idlh/81812.html, accessed on 20 November 2024). In humans, the inhibition of this biochemical pathway is used to prevent blood clotting, and therapeutic concentrations of warfarin lie in the range of 0.03–0.05 mg/kg [39]. Since warfarin has not been used in experiments with insects, we first tested the effect of this compound on flies’ survival in order to approach its toxicity. Warfarin does not cause the death of flies, as it does in vertebrate models, but on the contrary neutralises the negative effect of MSB and reduces the level of oxidative stress. In our experiments, warfarin in the tested concentrations did not significantly affect the number of flies emerging from pupae. This may indicate that the biochemical pathways of blood and haemolymph coagulation have diverged in evolution and need to be studied in more detail.

It is noteworthy that warfarin can be used in combination with MSB in human therapy. The authors of [40] used vitamin K therapy against the background of warfarin use. It was found that adding 150 mcg of vitamin K daily to warfarin therapy can lead to more stable anticoagulation in patients. Thus, sensitivity to warfarin is influenced by diets with different levels of vitamin K. Differences in human susceptibility to warfarin may be due to two factors: polymorphism of the *VKOR* gene (usually associated with increased resistance to warfarin) and polymorphism of the *CYP2C9* gene, which encodes a member of the cytochrome P450 family involved in the metabolism of warfarin. The high warfarin dose requirements of Africans compared to Asians, who require lower doses, is also related to the different distribution of *CYP2C9* variants [41]. It remains unclear how warfarin is degraded and eliminated from the Drosophila organism. According to BLAST (https://blast.ncbi.nlm.nih.gov/Blast.cgi?PROGRAM=blastp&PAGE_TYPE=BlastSearch&LINK_LOC=blasthome, accessed on 20 November 2024) analysis, the closest Drosophila *CYP2C9* homologue, *Cyp18a1*, has a 53% similarity to *CYP2C9* (for comparison, the similarity between *CYP2C9* and the mouse homologue is 88%). It is not known whether *Cyp18a1* is involved in warfarin detoxification. This needs to be investigated.

Notably, the similarity between human VKOR and its Drosophila homologue is also 53%. However, it is clear that the effects we obtained indicate that the function of Drosophila VKOR is blocked by warfarin. Thus, the low degree of similarity between Drosophila and human VKOR is not an indicator of their different functionality. Rather, individual conserved amino acid residues are important [42].

How can the Drosophila vitamin K cycle be used as a model to study the vitamin K cycle? Clearly, the Drosophila model has the advantage that warfarin does not have the same number of side effects as in vertebrates. In addition, other effects of warfarin that are not related to anticoagulation can be identified. Genes that respond to warfarin can be identified by analysing the transcriptomes of flies incubated on a medium containing warfarin. Transcriptomic studies can also help answer questions about the interaction of warfarin and vitamin K when they are used together. The selection of warfarin-resistant (sensitive) flies on a medium standardised for vitamin K content will help to identify genes and their variants that correlate with such resistance (sensitivity).

## 4. Materials and Methods

### 4.1. Fruit Fly Cultivation Conditions and Viability Testing for Vitamin K and Warfarin Levels

*D. melanogaster* wild-type Canton-S strain flies from the collection of the Department of Genetics of Moscow State University were kept at 25 °C on a standard nutrient agar medium. The chronic effect of MSB (“Vikasol”, Biosintez Sun Pharma, Mumbai, India) and warfarin (Warfarin STADA Arzneimittel, AG, Bad Vilbel, Germany) was studied by adding these agents to a standard nutrient agar medium.

First, we investigated the effect of different concentrations of MSB on this development. The concentrations of the MSB (100 nM–10 mM) were selected in accordance with a previously published study [14]. For this, adult flies were transferred onto a selected agar medium for egg laying, and after 24 h, they were removed. The eggs developed into adult flies, which were collected on the second day after hatching for further analysis. To study the acute effects of MSB and warfarin, two-day-old adults were placed for 24 h on paper soaked in a 5% (*w*/*v*) sucrose solution as a control and a 5% sucrose solution with the addition of 1 mM warfarin and 20 mM MSB. Sucrose was added to all media to encourage flies to drink water containing the tested substances. Second, we assumed that in Drosophila, as in vertebrates, the redox cycle of vitamin K should operate through the involvement of GGCX and VKOR. To test the contribution of VKOR, we used warfarin. As warfarin has not previously been used in Drosophila studies, we relied on previously obtained data on mammalian lethal doses of warfarin to select the doses. For mice, rats, cats, and dogs, the half-lethal dose is 1.5–6 mg/kg, and the IDLH (Immediately Dangerous to Life or Health) Concentration in humans is 100 mg/kg (https://www.cdc.gov/niosh/idlh/81812.html, accessed on 3 April 2024). In humans, the inhibition of this biochemical pathway is used to prevent blood clotting, and the therapeutic concentrations of warfarin lie in the range of 0.03–0.05 mg/kg [27]. We started to test the effect of warfarin by adding 0.1–10 mM warfarin (0.3–3 mg/mL) to the nutrient medium. Since larval development took place in the medium containing warfarin, we assumed that the concentration of warfarin in the larvae would eventually equal the concentration in the medium.

### 4.2. Extraction of Vitamin K from D. melanogaster Adult Flies for Mass Spectrometry Analysis

For one biological replicate, approximately 0.5–0.6 g of two-day-old adult flies were collected, snap-frozen in 1 mL of 100% cold (−20 °C) methanol containing 0.005% butylhydroxytoluene (BHT), and stored at −20 °C until the extraction was performed. All extraction stages were performed in the dark under red light. To obtain extracts, the flies were transferred with methanol to a ceramic mortar; ascorbic acid was added on the tip of a spatula to prevent oxidation and the flies were ground with a pestle until homogeneous. The homogenate was transferred to a 15 mL test tube, the mortar was washed 3 times with 500 μL of methanol with 0.005% BHT, and the extracts were combined. To pellet cell debris, the samples were centrifuged at 4000× *g* for 15 min and the supernatant was transferred to a fresh 15 mL tube. Vitamin K was further extracted by resuspension of a pellet in 1 mL of 100% methanol with 0.005% BHT and incubation for 15 min at room temperature, followed by centrifugation as described above. The last extraction step was repeated 2 more times. The final combined sample of all extracts was centrifuged for 15 min at 4000× *g*, and transferred to a new 15 mL tube. The resulting extract was lyophilised and stored at −20 °C until mass spectrometry analysis.

The extraction protocol for vitamin K using methyl tert-butyl ether (MTBE), ethyl acetate, and hexane–isopropanol (1:1, v:v) was the same as for methanol described above.

### 4.3. LC-MS Analysis of Vitamin K of Fly Extract

The LC-MS analysis was performed on an Agilent 1290 Infinity II HPLC system (Santa Clara, CA, USA), consisting of a four-channel high-pressure liquid pump, autosampler and column thermostat coupled online with an atmospheric pressure chemical ionisation (APCI) source to an AB Sciex TripleQuad 5500 mass spectrometer. Samples were separated on a C18 reversed-phase column (Zorbax Eclipse Plus C18 2.1 × 50 mm, 1.8 μm, Agilent, Santa Clara, CA, USA). Vitamin K_2_ (Sigma Aldrich, St. Louis, MO, USA) was used as a standard to optimise the overall protocol. As a result, isocratic separation conditions using methanol with water in a ratio of 95:5 at a flow rate of 0.5 mL/min as an eluent were selected as optimal. The column temperature was constant throughout the analysis and was set to 40 °C. The amount of vitamin K_2_ was quantified using diphenhydramine as an internal standard, which was added to a sample prior to the sample dissolution step. Detection of analytes in the chromatographic flow was performed in the multiple reaction monitoring (MRM) mode. The masses installed on the Q1 and Q3 quadrupoles were selected by analysing the fragment ion spectrum for the most intense and most characteristic fragment ions. The MRM mode parameters (collision energy (CE), input potential (EP), delustering potential (DP), and collision cell exit potential (CXP)) were selected by scanning the corresponding parameter in the maximum possible ranges (Supplementary Table S3). The ionisation source parameters were optimised by introducing a vitamin K_2_ standard sample through a T-splitter into the chromatographic line connected directly to the ionisation source. The corona current (NC)—5 μA, heater temperature (TEM)—500 °C, nebulizer gas pressure (GS1)—50 psi, and curtain gas pressure (CUR)—28 psi were optimised to achieve maximum signal intensity in MRM mode. The extracts were diluted to 15 mL with methanol, and 1 mL of the resulting solution was collected and evaporated to dryness in a vacuum centrifuge concentrator. The resulting dry sample was then redissolved in a mixture of methanol and water (95:5, v:v) and used for analysis.

### 4.4. Detection of ROS in D. melanogaster Homogenates by Electron Paramagnetic Resonance (EPR) Technique

Adult *D. melanogaster* flies were kept for 24 h in test tubes on filter paper soaked with a 5% (*v*/*w*) sucrose solution with the addition of the following additives: water (control), 20 mM MSB, 1 mM warfarin, and 20 mM MSB combined with 1 mM warfarin. For each sample, five males and five females were collected in a test tube.

For total oxidase capacity, we used a non-specific spin probe 1-hydroxy-4-isobutyramide-2,2,6,6-tetramethylpiperidinium chloride (TMT-H) (synthesised by Dr. N. N. Vorozhtsov Novosibirsk Institute of Organic Chemistry SB RAS, Novosibirsk, Russia). A total of 350 μL of 0.5 mM TMT-H solution was added to the Eppendorf tube, and the flies were homogenised with a pestle for 1 min and centrifuged at 5000× *g* for 5 min at 21 °C. A total of 250 μL of the supernatant was used for EPR analysis. The EPR spectra of TMT-H were collected on a RE-1307 EPR spectrometer (EPSI, Chernogolovka, Russia) at 21–22 °C, a microwave power of 22 mW, and a time constant of 0.1 s. Each characteristic spectrum is the result of five signal accumulations. The oxidative capacity of a sample was assessed from the intensity of the central component of the TMT-H EPR spectrum I0.

### 4.5. Isolation of Total RNA and cDNA Synthesis

Total RNA was isolated using the ExtractRNA kit (Evrogen, Moscow, Russia) according to the manufacturer’s protocol. For each sample, either 5 whole flies of each sex, or 20 adult heads of each sex, or 10 adult bodies (abdomen and thorax) of each sex were used. Deoxyribonuclease I (Thermo Scientific, Waltham, MA, USA) was used to purify RNA from DNA according to the manufacturer’s protocol. RNA quality was checked by electrophoresis in 1% agarose gel stained with ethidium bromide. RNA concentration was determined using the NanoDrop2000 instrument (Thermo Fisher Scientific, Waltham, MA, USA). The MMLV RT kit (Evrogen, Moscow, Russia) was used to synthesise cDNA from the matrix of the isolated total RNA according to the manufacturer’s protocol.

### 4.6. Real-Time PCR

A hot-start reagent kit containing Taq polymerase and the intercalating dye SYBR Green I (Evrogen, Moscow, Russia) was used for RT-PCR. The following primers were used: *hsp22*: forward 5′-CTTTCACGCCTTCTTCCAC-3′ and reverse 5′-GTGAGTTTGTAGCCATCCTTG-3′; GC: forward 5′-TACACTGCCACTTTCCACTG-3′ and reverse 5′-GAGCAGCAATGTTCCCACCA-3′. The reaction was carried out in a MiniOpticon Real-Time PCR System Amplifier (Bio-Rad Laboratories, Hercules, CA, USA). The following protocol was used: 5 min 95 °C, then 40 cycles with the following steps: melting—10 s at 95 °C, primer annealing—30 s at 55 °C, and synthesis—60 s at 72 °C. Deoxyribonuclease I-treated samples were used as a negative control. The normalisation of target gene expression was performed using three reference genes: *αTub84D* (forward 5′-GTGCATGTTGTCCAACACCAC-3′ and reverse 5′-AGAACTCTCCCTCCTCCATA-3′), *RPL40* (forward 5′-CTCGTGGTGGTATCATTG-3′ and reverse 5′-CAGGTTGTTGGTGTCC-3′), and *Elo-B* (forward 5′-GCACAAACATACACTCACG-3′ and reverse 5′-TTTCCTACTTCGCTTGCACC-3′). Statistical analysis was performed using GraphPad Prism 9; the two-tailed Mann–Whitney test was used to analyse the expression levels of the target genes.

## Data Availability

Additional information regarding the manuscript will be welcomed by the authors.

## Supplementary Materials

The following supporting information can be downloaded at: https://www.mdpi.com/article/10.3390/ijms26104808/s1.

## Abbreviations

The following abbreviations are used in this manuscript:

MSB	Menadione sodium bisulfite
GGCX	Gamma-glutamyl carboxylase
VKOR	Vitamin K epoxide reductase
MRM	Multiple reaction monitoring
EPR	Electron paramagnetic resonance
ROS	Reactive oxygen species
