# Supplementary material for: Effect of Warfarin on Lifespan and Oxidative Stress Tolerance of Drosophila melanogaster
Source: Int J Mol Sci. 2025 May 17;26(10):4808. doi: 10.3390/ijms26104808 (PMC12112360; doi:10.3390/ijms26104808)
Supplement: Supplementary file 1 [file ijms-26-04808-s001.zip › ijms-3587016-supplementary.pdf]

Table S1. Effect of warfarin alone and in combination with a lethal dose of MSB on larval viability and adult hatching of *D. melanogaster*.

Warfarin, mM	Presence of larvae and hatched adults	
	– 10mM MSB	+ 10mM MSB
0,0001	+	–
0,001	+	–
0,01	+	–
0,1	+	–
1	+	+
10	+	+

Table S2. Extractability of vitamin K_2 by organic solvents from fruit fly homogenates determined by multiple reaction monitoring (MRM).

Extraction agent	Methanol	MTBE	Hexane–isopropanol 1:1	Ethyl acetate
vitamin K, pg/ml	11	12	-	-

Table S3. MRM mode settings.

MRM transition	Q1	Q3	Dwell*, ms	DP, B	EP, B	CE, B	CXP, B
**VitK$_2$ quantifier**	445.4	187.2	50	89	10	33	21
**VitK$_2$ qualifier**	445.4	341.1	50	89	10	25	12
**DMD-IS**	256.3	152.1	50	55	10	20	14

*Dwell—Scan time of one MRM transition.
